# LOWER ESOPHAGEAL SPHINCTER PRESSURE MEASUREMENT UNDER STANDARDIZED INSPIRATORY MANEUVEURS

**DOI:** 10.1590/S0102-67202015000300007

**Published:** 2015

**Authors:** Jeany Borges e Silva RIBEIRO, Esther Cristina Arruda Oliveira DIÓGENES, Patrícia Carvalho BEZERRA, Tanila Aguiar Andrade COUTINHO, Cícera Geórgia Félix de ALMEIDA, Miguel Ângelo Nobre e SOUZA

**Affiliations:** Gastroenterology Laboratory, Ceará Federal University, Fortaleza, CE, Brazil

**Keywords:** Threshold maneuver, Esophagogastric junction pressure, Crural diaphragm, Gastroesophageal reflux, Lower esophageal sphincter

## Abstract

**Background:**

Through rhythmic variations, the diaphragm influence lower esophageal sphincter (LES) pressure acting as an external sphincter. LES pressure recording is characterized by increased pressure in inspiration due to contraction of the diaphragmatic crura that involves the sphincter.

**Aim:**

To describe a method of measuring LES pressure during standardized inspiratory maneuvers with increasing loads.

**Methods:**

The study population comprised of eight healthy female volunteers (average age of 31.5 years). An esophageal high-resolution manometry and impedance system was used for measuring the LES pressure during 3-second inspiratory efforts under 12, 24 and 48 cm H_2_O loads (Threshold maneuvers).

**Results:**

There was a significant difference between the average maximum LES pressure and the average maximum basal LES pressure during the first (76.19±17.92 difference, p=0.0008), second (86.92±19.01 difference, p=0.0004), and third seconds of the maneuver (90.86±17.93 difference, p=0.0002), with 12, 24 and 48 cmH_2_O loads.

**Conclusion:**

This maneuver is a standardization of the inspiratory LES pressure and may better differentiate patients with reflux disease from healthy individuals, and may also be useful for monitoring the treatment of these patients through inspiratory muscle training.

## Abstract (pt)

**Racional:**

Através de variações rítmicas, o diafragma influencia a pressão do EEI, atuando como um esfíncter externo. O registro manométrico da sua pressão caracteriza-se por aumento de pressão na inspiração resultante da contração da crura diafragmática que envolve o esfíncter.

**Objetivo:**

Descrever um método de medida da pressão do esfíncter esofágico inferior (EEI) durante manobras inspiratórias padronizadas, com cargas crescentes.

**Métodos:**

Oito voluntários sadios (sexo feminino, média de idade de 31,5 anos) participaram do estudo. Uma manometria esofágica de alta resolução e impedanciometria mediram a pressão do EEI durante manobras inspiratórias com o Threshold sob cargas de 12, 24 e 48 cm H_2_O.

**Resultados:**

Comparando-se as médias houve diferença significativa entre a pressão máxima do EEI e a sua pressão basal máxima durante o primeiro (diferença de 76,19±17,92, p=0,0008), segundo (diferença 86,92±19,01, p=0,0004) e terceiro segundos da manobra (diferença 90,86±17,93, p=0,0002), tanto com carga de 12 cm de H_2_O, quanto com 24 e 48 cm.

**Conclusão:**

Esta manobra é uma padronização da pressão inspiratória do EEI e pode diferenciar melhor pacientes com doença do refluxo de indivíduos sadios, podendo também ser útil na monitorização do tratamento desses pacientes por meio do treinamento muscular inspiratório.

## INTRODUCTION

The lower esophageal sphincter is a 2-4 cm high pressure zone with intra-abdominal and intra-thoracic segments. The pressure inversion point separates the lower esophageal sphincter (LES) segments and the intra-thoracic yields a negative pressure during inhalation. This segment is approximately 0.5 cm long, generally located in the middle of the high pressure zone, and related to the crural diaphragm. The LES normally extends two to four centimeters distal to the inversion point and corresponds to the intra-abdominal segment. The high pressure zone is asymmetrical with the left lateral and posterior pressures significantly higher 7 .

The diaphragm influences rhythmically the LES pressure, acting as an external sphincter. Its manometric profile is represented by an increase in pressure during inhalation, due to the contraction of the diaphragm that skirts the esophagus 7 .

There is evidence that the inspiratory pressure better distinguishes reflux esophagitis patients from controls 8 . Some patients with esophagitis may not increase inspiratory LES pressure during inhalation as much as controls 8 and some adaption may occur in other conditions 9 . The diaphragm contraction squeezes and lowers the gastroesophageal junction during inspiration.

The topographic profiles of the gastroesophageal juntion delivered by high resolution manometry (HRM) shows both its pressure and lowering.

The aims of this study was to describe a measurement method of the LES pressure during standardized inspiratory maneuvers with stepwise increasing efforts.

## METHODS

### Volunteers

Eight healthy volunteers (without GERD symptoms) were submitted to HRM of the esophagus in the Gastroenterology Research Laboratory of the Clinical Medicine Department of the Ceará Federal University (Fortaleza, CE, Brazil). The study was a qualitative and quantitative in humans. The ethical principles concerning human research set by the 196/96 resolution of National Health Council was followed.

### Manometry

HRM is a standard method of measuring the pressure gradient across the gastroesophageal junction and its relaxation, allowing accurate diagnosis of diseases associated with hyper or hypotonicity. It also allows the localization of the LES upper border which is necessary for pH monitoring probe positioning 4 .

HRM was performed with volunteers in the supine position and after at least 6 h fasting. The probe had 36 pressure 1 cm apart and 18 impedance sensors 2 cm apart (Given Imaging, Yokneam, Israel). Pressure calibration was performed at 0 and 300 mmHg and zeroed to atmospheric pressure before the procedure. The probe was positioned through one nostril with at least five sensors distal to the diaphragm. The protocol included a basal period, six 5-ml saline swallows and a respiratory maneuver 2 .

### Inspiratory maneuver

**FIGURE 1:**
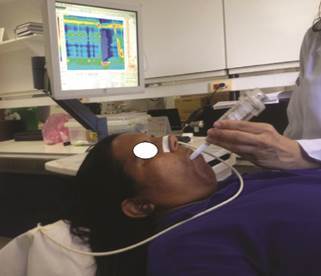
Volunteer performing an inspiratory maneuver with the Threshold device

The respiratory maneuver has been previously descried 2 . The volunteers stayed at the supine position without swallowing and the LES pressure was measured for 20 seconds before and during the maneuver. The maneuver consisted of fast and forced inhalation through a membrane valve (Threshold IMT, Philips Respironics, Andover, MA, USA) whose closing pressure (cmH_2_O) could be adjusted by a compression spring ( Figure 1 )^1^. All volunteers trained the maneuvers and undertook inhalations with 12, 24, and 48 cmH_2_O. 

### Data analysis

**FIGURE 2:**
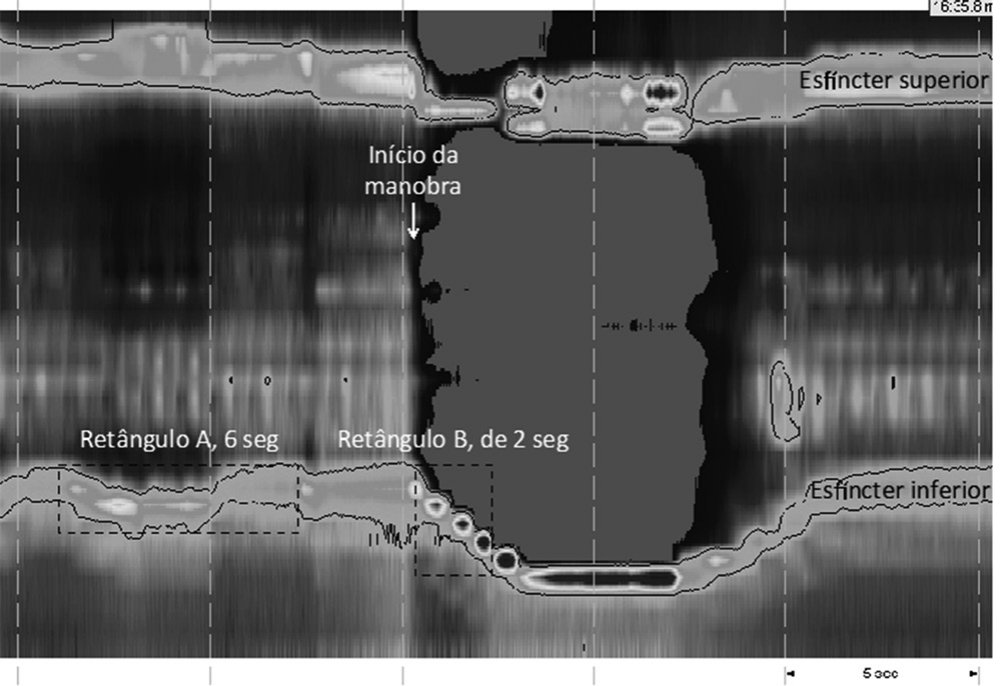
Variables obtained during the Threshold maneuver

*Variable definition (* Figure 2

The following variables were measure with the ManoView Analysis Software 3.0, with 20 mmHg isobaric pressure.

The average basal pressure (mmHg) was the mean pressure of the "A" rectangle with the height defined by the proximal and distal borders of the LES and the length equal to six seconds. 

The basal contractility integral (IC) was the product pressure x height x length of the "A" rectangle inside the 20 mmHg isobaric contour.

The minimal inspiratory oral pressure (mmHg) was the lowest hypopharyngeal pressure during the maneuver.

The minimal inspiratory esophageal pressure (mmHg) was the lowest esophageal pressure during the maneuver.

The LES lowering was figured out on the "B" rectangle, defined by two angles, first, the right angle formed by the horizontal line passing through the upper border of the LES and the vertical line at the start of the maneuver. The second angle was formed by a vertical line at a "t" time and the LES distal border at time t. The LES lowering (ds) was the height of the rectangle B (cm).

The maximal LES pressure (mmHg) was the highest pressure in rectangle B at each time t.

The LES contractility integral (CI) was the product pressure x height x length of the "B" rectangle inside the 20 mmHg isobaric contour at each time t.

Time assumed value of t equal to 1, 2, and 3 seconds.

Groups of similar variables were tested for distribution with the Student t test at a significance cutoff of 0.05.

## RESULTS

 Eight female healthy volunteers aged 31.5 years in average (21-47 years) and average BMI of 24.28 kg/m^2^ (17.3-30.61 kg/m^2)^ were studied.

The LES average basal pressure before the maneuver with 12 cmH_2_O load was 61.61±17.63. The basal CI was 198.91±117.92 ( Table 1 ).

During the 12 cmH_2_O maneuver, there was a stepwise lowering of the diaphragm (DS) across the first, second, and third seconds (5.3±0.78 cm, 5.9±0.89 cm, and 6.06±0.98 cm, respectively). The DS rate decreased across the maneuver (5.48±0.77 cm/s at 1s, 2.95±0.46 cm/s at 2s, and 2.01± 0.33 cm/s at 3s). On the other hand, the maximal pressures and the CI increased across the maneuver ( Table 1 ). The LES maximal pressures increment were higher across this maneuver (1s: 76.19±17.92, p=0.0008; 2s: 86.92±19.01, p=0.0004; 3s: 90.86±17.93, p=0.0002). Similar results were obtained for 24 and 48 cmH_2_O ( Tables 2 and ^3^ ), except for the third second of 24 cmH_2_O load.

**TABLE 1:** LES variables before and during the maneuver with 12 cmH_2_O load

Parameters	Mean±SD	Variation
Basal	Maximal LES pressure	61.61 ± 17.63	38.4 - 93.5
	Basal LES CI	198.91 ± 117.92	70.6 - 428.2
	Minimal oral pressure	-16.15 ± 8.38	-36.2 - (-11.8)
	Minimal esophageal pressure	-41.92 ± 18.74	-86.9 - (-28.6)
	Average intragastric pressure	16.22 ± 4.06	11.4 - 24.4
Maneuver time	
1 second	DS	5.3 ± 0.78	4.4 - 6.4
	DS/DT	5.48 ± 0.77	4.6 - 6.5
	Maximal LES pressure	137.8± 47.51	79.7 - 225.5*
	LES CI	101.87 ± 46.67	32 - 147
2 seconds	DS	5.9 ± 0.89	4.9 - 7.6
	DS/DT	2.95 ± 0.46	2.4 - 3.8
	Maximal LES pressure	148.53 ± 50.81	80.5 - 241.2^#^
	LES CI	220.78 ± 114.64	68.6 - 418.8
3 seconds	DS	6.06 ± 0.98	4.9 - 8
	DS/DT	2.01 ± 0.33	1.6 - 2.7
	Maximal LES pressure	152.47 ± 47.56	92.2 - 241.2^&^
LES CI	328.18 ± 167.1	133 - 627.1

LES=lower esophageal sphincter; CI=contractility integral; DS=diaphragm lowering; DS/DT=DS rate; SD=standard deviation.* p=0.0008; ^#^p=0.0004, ^&^p= 0.0002, versus maximal LES pressure (Student t test).

**TABLE 2:** LES variables before and during the maneuver with 24 cmH_2_O load

Parameters	Mean±SD	Variation
Basal	Maximal LES pressure	64,32 ± 14,29	49 - 93,5
	Basal LES CI	252,81 ± 150,04	105,5-489,6
	Minimal oral pressure	-23,57 ± 8,21	-43,2-(-17,1)
	Minimal esophageal pressure	-48,8 ± 32,14	-128-(-33,9)
	Average intragastric pressure	17,5±5,87	10,5-27
Maneuver time	
1 second	DS	4,77 ± 0,85	3,9-6,3
	DS/DT	4,8 ± 0,84	3,9 - 6,3
	Maximal LES pressure	134,42 ± 32,18	88-168*
	LES CI	105,5 ± 38,63	46,7- 149,5
2 seconds	DS	5,62 ± 0,93	4,6 - 7,2
	DS/DT	2,75 ± 0,47	2,3-3,6
	Maximal LES pressure	152,34 ± 35,09	101,1-207,3^#^
	LES CI	217,11 ± 75,4	103-317,9
3 seconds	DS	5,74 ± 1,09	4,6-7,8
	DS/DT	1,9 ± 0,37	1,56 - 2,6
	Maximal LES pressure	125,06 ± 69,14	-29,6-188,9^&^
LES CI	333,91 ± 124,6	175,8-500,3

LES=lower esophageal sphincter; CI=contractility integral; DS=diaphragm lowering; DS/DT=DS rate; SD=standard deviation. * p=0.0001; ^#^p<0.0001, ^&^p= 0.029, versus maximal LES pressure (Student t test).

**TABLE 3:** LES variables before and during the maneuver with 48 cmH_2_O load

Parameters	Mean±SD	Variation
Basal	Maximal LES pressure	56,56±17,93	26,6-86,7
	Basal LES CI	184,36±136	-87-321
	Minimal oral pressure	-39,5 ± 36,89	-51,3- (-31,5)
	Minimal esophageal pressure	-55,77 ± 29,77	-128,8 - (-37,7)
	Average intragastric pressure	17,45 ± 6,32	10-30,2
Maneuver time	
1 second	DS	4,3 ± 1,25	2,7-6,2
	DS/DT	4,43 ± 1,31	2,7-6,5
	Maximal LES pressure	136,79 ± 33,97	94,9-190,9*
	LES CI	104,3 ± 49,8	44-175,5
2 seconds	DS	4,8 ± 1,43	2,6-7,3
	DS/DT	2,4 ± 0,71	1,3- 3,6
	Maximal LES pressure	140,95 ± 33,95	94,9-192,5^#^
	LES CI	206,63 ± 79,35	118,4-326,1
3 seconds	DS	5,28 ± 1,33	3,3-7,6
	DS/DT	1,75 ± 0,44	1,1-2,5
	Maximal LES pressure	150,76 ± 40,41	94,9-208,7^&^
LES CI	312,19 ± 105,47	188,7-487,1

LES=lower esophageal sphincter; CI=contractility integral; DS=diaphragm lowering; DS/DT=DS rate; SD=standard deviation. * p<0.0001; ^#^p<0.0001, ^&^p< 0.001, versus maximal LES pressure (Student t test).

**FIGURE 3:**
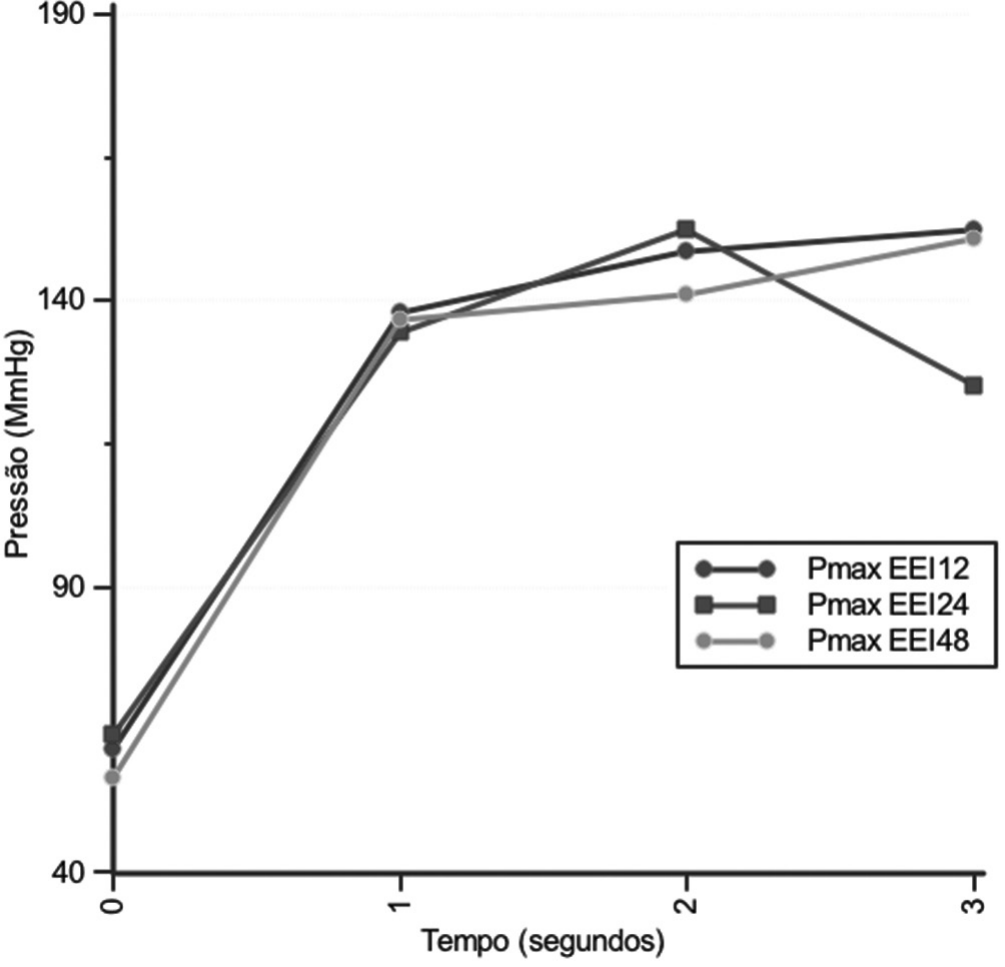
LES pressure before and during the inspiratory maneuvers with loads of 12 cmH_2_O, 24 cmH_2_O, and 48 cmH_2_O at 1, 2, and 3 seconds

Figure 3 shows the increase in LES pressure across the three loads of the maneuvers.

## DISCUSSION

HRM allows real time analysis of the LES pressure and the components of the anti-barrier reflux. Then, it is an important tool for the pathophysiology study of GERD 6 .

The crural diaphragm contractions are also related to shifts in gastroesophageal junction pressure. Generally, these contractions are associated with respiration. There is a 10 to 20 mmHg increase in pressure during normal inhalation, and 50 to 150 mmHg during deep inspiration 5 . The Threshold device is used to strengthen the inspiratory muscles. It is made by one-way diaphragm valve kept in place by a spring which compression can be adjusted manually. Therefore, the inspiratory effort necessary to displace the membrane and allow airflow can be increased linearly. Inspiratory muscles are trained this way 3 10 . There is no effort during exhalation.

This inspiratory maneuver has been adapted to HRM in order to measure the gastroesophageal junction inspiratory pressure in GERD 8 9 . 

During the 12 cmH_2_O maneuver, there was a stepwise increase in the maximal inspiratory LES pressure, relative to the maximal basal pressure - 2.23 times at the first second, 2.41 times at the second second, and 2.47 times at the third second. These relative increments were 2.09 times at the first second, 2.3 times at the second second, and 1.94 times at the third second for the 24 cmH_2_O maneuver, and 2.41 times at the first second, 2.49 times at the second second, and 2.66 times at the third second for the 48 cmH_2_O maneuver ( Table 3 ).

The inspiratory maneuver depends on volunteer effort and collaboration. This is a weakness of this method as can be seen by the large variation of the maximal inspiratory LES pressure values. The relatively lower increase in the maximal pressure during the third second of the 24 cmH_2_O maneuver could have occurred because of this drawback. On the other hand, the stepwise increase in LES pressure across the 1, 2, and 3 seconds of maneuver is a strong-point of the method. This attribute may help differentiate distinct pathological conditions.

## CONCLUSION

This maneuver is a standardization of the LES inspiratory pressure measurement. It may better differentiate GERD patients from healthy individuals, since the former ones may have a crural deficiency and a lower inspiratory LES pressure. Furthermore, this maneuver may help monitor the efficiency of inspiratory muscle training for GERD 8 .
